# Action of *Coriandrum sativum* L. Essential Oil upon Oral *Candida albicans* Biofilm Formation

**DOI:** 10.1155/2011/985832

**Published:** 2011-05-21

**Authors:** V. F. Furletti, I. P. Teixeira, G. Obando-Pereda, R. C. Mardegan, A. Sartoratto, G. M. Figueira, R. M. T. Duarte, V. L. G. Rehder, M. C. T. Duarte, J. F. Höfling

**Affiliations:** ^1^Department of Microbiology and Immunology, Dental School of Piracicaba, University of Campinas-UNICAMP, 13414-903 Piracicaba, SP, Brazil; ^2^Research Center for Chemistry, Biology and Agriculture, University of Campinas-UNICAMP, P.O. Box 6171, 13083-970 Campinas, SP, Brazil

## Abstract

The efficacy of extracts and essential oils from *Allium tuberosum, Coriandrum sativum, Cymbopogon martini, Cymbopogon winterianus,* and *Santolina chamaecyparissus* was evaluated against *Candida* spp. isolates from the oral cavity of patients with periodontal disease. The most active oil was fractionated and tested against *C. albicans* biofilm formation. The oils were obtained by water-distillation and the extracts were prepared with macerated dried plant material. The Minimal Inhibitory Concentration—MIC was determined by the microdilution method. Chemical characterization of oil constituents was performed using Gas Chromatography and Mass Spectrometry (GC-MS). C. sativum activity oil upon cell and biofilm morphology was evaluated by Scanning Electron Microscopy (SEM). The best activities against planktonic *Candida* spp. were observed for the essential oil and the grouped F_8–10_ fractions from *C. sativum*. The crude oil also affected the biofilm formation in *C. albicans* causing a decrease in the biofilm growth. Chemical analysis of the F_8–10_ fractions detected as major active compounds, 2-hexen-1-ol, 3-hexen-1-ol and cyclodecane. Standards of these compounds tested grouped provided a stronger activity than the oil suggesting a synergistic action from the major oil constituents. The activity of *C. sativum* oil demonstrates its potential for a new natural antifungal formulation.

## 1. Introduction

During the last two decades, the yeasts of the genus *Candida* have received notable attention in medical literature. Colombo and Guimarães [1] emphasized the increase of nosocomial infections caused by *Candida* spp. In the beginning of the 80s, *Candida* spp. were the seventh most frequent pathogen associated with hospital*-*acquired infections in the United States of America. At the end of the 80s, this agent represented the fifth most frequent pathogen and presently, according to the author, accounts for 8% of the cases of hospital*-*acquired infections and is considered the fourth main isolated pathogen present in diagnostic tests. 

The frequency of commensal *Candida* species isolated in the oral cavity of clinically healthy individuals varies from 40 to 60% [2]. Several factors predispose to the development of candidiasis, such as endocrine disorders, pregnancy, iron deficiency, immune disorders, and xerostomy [3]. The occurrence of oral candidosis can be related to the host and to the yeast itself. The most cited causes related to the host are diabetes mellitus (DM), loss of teeth, changes in dietary habits, oral hygiene, systemic diseases, hormonal changes, immunosuppression, radiation, and chemotherapy [4]. Among the factors related to the yeast (also called virulence factors), adhesion phenomenon, mycelium formation, phenotypic variability, production of extracellular enzymes (protease and phospholipase), and toxins [4–6] can be observed. Furthermore, the hability of the *Candida* species to grow as a biofilm can increase the resistance to drugs, blocking or preventing the access of antifungals to the more internal biofilm structure [7].

Candidosis is considered the most common fungal infection of the human oral cavity, and *C. albicans* is the main species related although nonalbicans species such as *C. tropicalis, C. parapsilosis, C. krusei, C. glabrata*, and* C. dubliniensis,* are becoming common among certain groups of patients [3, 8–10]. The occurrence of severe periodontitis has also been associated with the isolation of species of *Candida* spp. from periodontal lesions [11–14]. The yeast, through the access to tissues, produces metabolites that lead to inflammation.

The widespread use of azole antifungals for the treatment of oral candidosis, especially after the AIDS era, seems to have been the predominant factor for the increase in frequency of non-*Candida albicans* species, especially those naturally more resistant to this class of antifungal agents, such as *C. glabrata* and *C. krusei* [15]. Data from some studies suggest that prolonged or intermittent treatment with antifungal agents modifies *C. albicans* prevalence, increasing the frequency of other *Candida* species [16, 17]. 

The resistance of human pathogens to multiple drugs is well known today and indicates the need to search for antimicrobial substances from new sources, including plants used in folk medicine, which present a great variety of compounds with therapeutic properties. The use of plants as a source of drugs is prevalent in developing countries as an alternative to health problems and is well established in some cultures and traditions, especially in Asia, Latin America, and Africa [18]. Many native plants in these regions have not yet been studied and can be researched for antimicrobial action, in contrast to European native plants that have already been exhaustively studied [19]. According to Duarte & Figueira [19], the information available on medicinal plants active against *C. albicans* has not, until recently, resulted in effective formulations for human or animal use, except for some patents concerning material derived from the plant families *Allium* [20], *Radix gentianae* [21], and five extracts studied by Lee et al. [22].

In Brazil, despite the existing rich flora and biodiversity, data from only approximately 44 species and 22 families with positive activities are available, including native and exotic plants [19]. The aim of this study was to evaluate the antimicrobial activity of extracts, essential oils, and fractions from medicinal plants belonging to the CPMA-Medicinal and Aromatic Plant Collection at CPQBA/UNICAMP, against planktonic cells of *Candida* spp. from CBS collection and isolates from patients with periodontal disease. The most active oil, from *Coriandrum sativum *L, was tested against *C. albicans* biofilm formation. 

## 2. Materials and Methods

### 2.1. Samples

For the development of this research, *Candida* spp. reference strains obtained from Netherlands Collection—CBS (*C. albicans* CBS 562, *C. dubliniensis* CBS 7987, *C. krusei* CBS 573, *C. tropicalis* CBS 94, and *C. parapsilosis* CBS 604) and clinical isolates from volunteers of the Dentistry Faculty of Piracicaba-FOP/UNICAMP, São Paulo-Brazil as *C. albicans* (3A5, 13A5, 41M2, and 50M2), *C. dubliniensis* 26A2, 26A3, and 26A4), *C. krusei* (56M3, 56M4, and 56M5), *C. tropicalis* (53M6, 52M7, and 53M8), and *C. parapsilosis* (45M2, 45M4, and 45M5) were used. The isolates were collected from oral mucosa and were identified on chromogenic medium CHROMagar-*Candida* and by morphological and biochemical tests. The codes of the clinical isolates correspond to the number of the volunteers and the place of origin (A from periodontal pocket and M from mucosa). Subsequently, the yeasts were stored at −70°C in YPD medium with 15% glycerol and maintained at the mycology collection of the Department of Microbiology and Immunology from FOP/UNICAMP. This research was performed under the authorization of the Research Ethics Committee (008/2003). 

### 2.2. Medicinal Plants

The plants studied as *Allium tuberosum* Rottl. ex Spreng, *Coriandrum sativum* L, *Cymbopogon martinii* (Roxb.) J. F. Watson, *C. winterianus* Jowitt, and Santolina chamaecyparissus were grown in the experimental field of the Research Center for Chemistry, Biology and Agriculture (CPQBA), University of Campinas, São Paulo, Brazil. The plants were collected from November 2001 to November 2002, during the morning after dewpoint. Voucher specimens were deposited at the University of Campinas Herbarium (UEC) and identified by Dr. Washington Marcondes Ferreira Neto (curator), or at the herbarium at the CPQBA. 

### 2.3. Essential Oil Extraction

The essential oils were obtained from 100 g of aerial fresh plant parts by water distillation using a Clevenger-type system for 3 h. The aqueous phase was extracted three times with 50 mL of dichloromethane. The pooled organic phases were dried with sodium sulphate and filtered, and the solvent was evaporated until dryness. Oil samples were stored at −25°C in sealed glass vials [23]. 

### 2.4. Extracts Preparation

Dried plant material (10 g) was macerated with 150 mL of hexane or dichloromethane, as indicated, and submitted to shaking at 200 rpm at room temperature for 3 h. Subsequently, the extracts were filtered, and the plant residues were re-extracted with fresh 70% ethanol. The pooled filtrates were concentrated under vacuum and stored at 4°C until further use [23]. 

### 2.5. Essential Oil Fractionation

 The essential oil that presented best activity was fractioned in a dry column prepared with silica gel 60 Merck (0,063–0,200 mm). A cellulose bag (3 cm diameter) was used for support. The essential oil was put directly into the packed column and eluted. The mobile phase utilized in each column was defined after essential oil analysis using TLC (thin layer chromatography), in different eluents. After elution, the column was fractioned into three parts, and the fractions were transferred individually to the appropriate vials, solubilized in ethyl acetate, and analyzed by TLC. Similar fractions were grouped and filtered using vacuum and the silica residues extracted with ethyl acetate [23]. 

### 2.6. Activity Assay-Minimal Inhibitory Concentration (MIC) Test

Yeasts were grown overnight at 36°C in Sabouraud Dextrose Agar (Merck) plates. Inocula for the assays were prepared by diluting scraped cell mass into 0.85% NaCl solution, adjusted to McFarland scale 0.5, and confirmed by spectrophotometric reading at 580 nm. Cell suspensions were finally diluted to 10^4^ UFC mL^−1^ in RPMI-1640 medium (Difco) for assays. MIC tests were carried out according to CLSI [24], using tissue culture test plate (96 wells), containing 100 *μ*L RPMI-1640 medium. The stock solutions of the essential oil or crude extracts obtained from medicinal plants were diluted and transferred into the first well, and serial dilutions were performed to obtain concentrations ranging from 1 to 0.03 mg mL^−1^. Nystatin and Fluconazol (Merck) were used as antimycotic control reference ranging from 1 to 0.03 mg mL^−1^. The yeast inoculum was added to all wells, and plates were incubated at 36°C for 48 h. MIC was defined as the lowest concentration of oil or extract that inhibited visible growth, as indicated by the RPMI-1640 medium color change (pink to yellow). 

### 2.7. *C. albicans* Biofilm Induction

Standardized *C. albicans* cells (1.0 × 10^6^ cells/mL in RPMI 1640 medium) were introduced into wells of 96 well tissue culture plates (TPP) containing RPMI-1640 medium with 2% sucrose and were incubated for 1.5 h at 37°C. After the 1.5 h initial adhesion, the medium was aspirated, and nonadhered cells were removed and washed with sterile distilled water. RPMI 1640 medium was added to the adhered cells in the wells. The plates were incubated for 24 h at 37°C [25]. 

### 2.8. Activity of the Essential Oil and Fractions from *C. sativum* on the Biofilm Formation in *C. albicans*

To evaluate the antimicrobial activity of *C. sativum* essential oil on *C. albicans* biofilm formation, the samples were applied at regular intervals during 24 h at two different concentrations, corresponding to the MIC previously found for the planktonic cells, and 1 mg/mL. The applications were carried out before planktonic cells adhesion (*T*_0_), after preadhesion (*T*_3_ h) and after 6 h, 12 h, 18 h, 24 h, and 48 h. The plates were read at the following interval after application. The controls fluconazole (fungistatic)—0064 mg/mL and nystatin (fungicidal)—1 mg/mL were also included in the tests. The tests were performed in three independent experiments, each in triplicate [25]. 

### 2.9. Biofilm Quantification

The biofilm developed in each well was washed twice with 200 *μ*L of distilled water and then dried for 45 min. In each well, 110 *μ*L of 0.4% crystal violet were added for 45 min. After this procedure, the wells were washed four times with distilled water and immediately discolored with 350 *μ*L of 95% ethanol. Elapsed 45 minutes after the last procedure, 100 *μ*L of discolored solution was transferred to a well of a new plate and the crystal violet measured at 595 nm in an ELISA reader (SpectraMax 340 tunable MICROPLATES Reader, Molecular Devices Ltda). The amount of biofilm formed was measured, subtracting the absorbance from the absorbance values of the control wells [26]. 

### 2.10. Scanning Electron Microscopy (SEM)

In order to evaluate *C. albicans* integrity cells using SEM, the biofilms were first developed in Lab-Tek chambered coverglass (Nunc), as described above, and treated with *C. sativum* essential oil and standard drugs. The samples were fixed in glutaraldehyde 0.15 M 2.5% (v/v) PBS for one hour at room temperature. After this procedure, the chambered coverglasses were treated with 1% osmium trioxide for one hour and washed three times with 3 mL of distilled water. The chambered coverglasses were then hydrated with an ethanol series (50% to 100%), dried to a critical point and coated with gold in the metalizer, and then observed using a scanning electron microscope [27]. 

### 2.11. Gas Chromatography and Mass Spectrometry Analyses (GC-MS)

The identification of volatile constituents was performed using a Hewlett-Packard 6890 gas chromatograph, equipped with an HP-5975 mass selective detector and HP-5 capillary column (25 m × 0.25 mm × 0.25 *μ*m diameter). GC and GC-MS were performed using split/splitless injection, with the injector set at 220°C, column set at 60°C, with a heating ramp of 3°C min^−1^ and a final temperature of 240°C, and the MS and FID detector set at 250°C. Helium was used as a carrier gas at 1 mL min^−1^. The GC-MS electron ionization system was set at 70 eV. A sample of the essential oil was solubilized in ethyl acetate for the analyses. Retention indices (RIs) were determined by coinjection of hydrocarbon standards. The oil components were identified by comparison with data from the literature, the profiles, the Nist 05 library, and by co-injection of authentic standards, when available [28]. 

### 2.12. Statistical Analysis

All statistical tests were performed with one-way ANOVA, and *P* value of <.05 was considered statically significant. The statistical test was run using STATISTICA v.5.0 (Stasoft, USA) system software, and graphics were carried out using SIGMAPLOT v.10.0. System software (SPSS Inc.). Different treatments were compared at all times during the biofilm assay. 

## 3. Results

### 3.1. Oil and Extracts Yields

Oil and extracts yields were expressed in relation to dry weight of the plant material (% w/w). Larger quantities of essential oil were obtained from *C. martinii* (1.36%) and *C. winterianus* (1.88%) followed by *S. chamaecyparissus* (0.15%), *C. sativum* (0.08%), and *A. tuberosum* (0.11%). Compared to essential oils, extract yields were higher and ranged from 1,33% (*C. sativum*) to 18.5% (*A. tuberosum*). The intermediate yields were 7.33% (*C. martini*), 10.14% (*S. chamaecyparissus*), and 14.68% (*C. winterianus)*. 

According to the activity results, the *C. sativum* essential oil was fractioned in a dry column and the fractions grouped according to the equivalence of the bands presented in thin layer chromatography (TLC) analysis. The grouped *F*_8–10_ fractions presented the highest yield (155.20 mg/g) followed by F7 fraction (120.40 mg/g), F5 fraction (25.90 mg/g), F6 (22.60 mg/g), and F3-4 grouped fraction (8.74 m/g). 

### 3.2. Essential Oil and Extract Activity against Planktonic * Candida * spp

Minimum inhibitory concentration (MIC) of the essential oils (EOs), dichloromethanic extracts (DEs), and hexanic extract (HE) were tested against *Candida* spp. planktonic cells, as indicated in Table 1. In general, the oils and extracts presented a good action against planktonic *Candida* spp. and clinical isolates; however, the oil and the hexanic extract from *C. sativum* were capable of inhibiting all strains at MIC values between 0.015–0.500 mg/mL and 0.007–1.000 mg/mL. 

The activity of the fractions of *C. sativum* essential oil was also tested against the planktonic cells of *Candida *spp., presenting in general strong activity, except for fraction 5 (Table 2). The lowest activity was observed for grouped *F*_8–10_ *MIC value*, the most polar fraction found by TLC analysis. In order to compare the results of inhibition obtained from the medicinal plants, two commercially available antifungal agents were chosen: nystatin (polienic fungicide) and fluconazole (azole fungistatic). 

### 3.3. *C. sativum* Essential Oil Activity against *C. albicans* Biofilm

**Figure 1:**
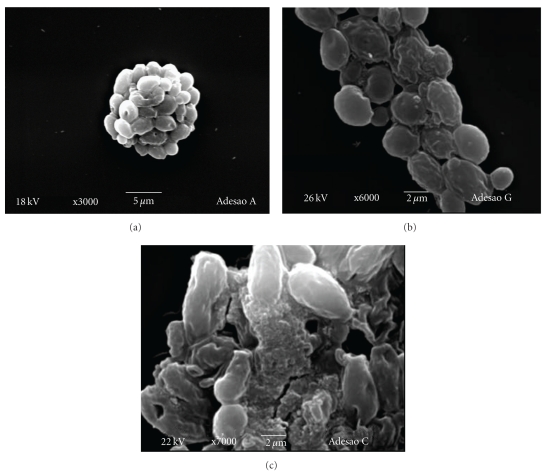
Biofilm kinetic development: (a) Control, without treatment; (b) action of nystatin, fluconazole and *C. sativum *essential oil in the biofilm kinetic development for *C. albicans* CBS 562; (c) action of nystatin, fluconazole and *C. sativum *essential oil in the biofilm kinetic development for *C. albicans* clinical isolate 13A5.

Figure 1(a) shows the kinetic biofilm development for *C. albicans* CBS 562 and clinical isolate 13A5. The action of nystatin, fluconazole, and *C. sativum *essential oil upon biofilm growth was presented in Figures 1(b) and 1(c). The crude essential oil from *C. sativum* and drugs was applied at regular intervals during biofilm cultivation. The results shown in Figures 1(b) and 1(c) indicate a clear effect of the oil on biofilm formation, characterized due to an increase in the lag phase and a decrease in the biofilm growth. Similar effects were observed for nistatin, though inferior to fluconazole (*P* ≤ .05).

The statistical analysis demonstrated a significative difference (*P* ≤ .05) after 48 h of treatment (Figures 1(b), 1(c)). Considering the results obtained for *C. albicans* CBS 562, the best treatments were nystatin (1 mg/mL) and essential oil (0.125 mg/mL). For the clinical isolate 13A5, the effect on the biofilm was statistically identical as that for nystatin (1 mg/mL), fluconazole (0.0641 mg/mL), and essential oil (1 mg/mL), though inferior for the essential oil at 0.125 mg/mL. 

### 3.4. Chemical Characterization of Oil Constituents

The chemical composition of the essential oil and grouped *F*_8–10_ fractions from *C. sativum* is shown in Table 3. The evaluation was performed by gas chromatography coupled to mass spectrometry (GC-MS). The oil analysis indicated the presence of volatile derivatives of alcohols and aldehydes, such as decene, decanal, decenol, decenal, decanol, dodecanal, dodecanol, tetradecenal, tetradecanol, tridecanol, hexenol, and cyclodecane. Fraction *F*_8–10_ was enriched with 3-hexen-1-ol (16.08%), 2-hexen-1-ol (14.52%), and 1-decanol (44.16%). Further experiments were carried out aiming to evaluate the action of the isolated components (Sigma-Aldrich standards) against *C. albicans* planktonic cells (Table 4). 

### 3.5. Scanning Electron Microscopy

**Figure 2:**
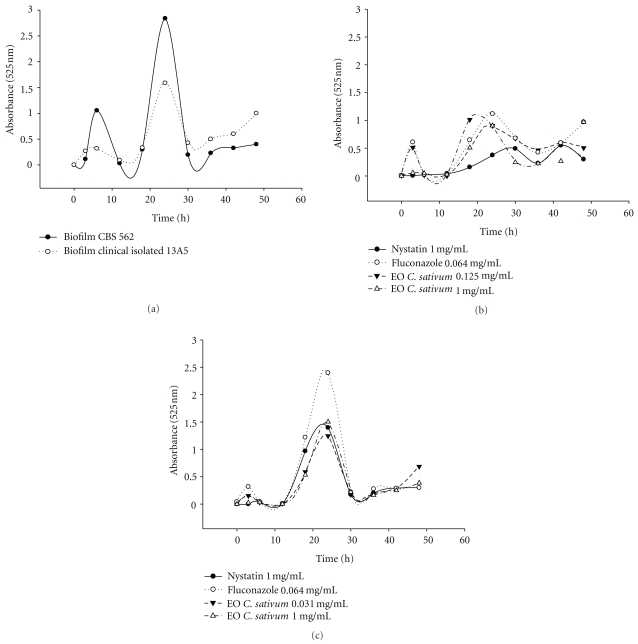
Scanning electron microscopy (SEM) of the *C. albicans* biofilm: (a) without treatment; (b) treated with *C. sativum* crude essential oil; (c) treated with grouped fractions *F*_8–10_.

The action of *C. sativum* crude oil and grouped *F*_8–10_ fractions upon *C. albicans* cells and biofilm morphology, evaluated by scanning electron microscopy (SEM), is presented in Figures 2(a)–2(c) and demonstrates that the plant materials caused an amendment in cell structure. 

## 4. Discussion

The activity of natural products upon microorganisms has been recently confirmed by literature. Plant materials such as extracts, essential oils, and isolated compounds have demonstrated inhibition against bacteria [29], yeast [30], and filamentous fungi [31]. Most studies, however, focus on planktonic cells; nevertheless, the fact that several microorganisms are capable of growing as a biofilm, leading to a greater resistance to drugs, indicates the need for further activity studies with these natural products regarding biofilm mechanism formation. In the present study, essential oils and extracts from 5 different medicinal species, with previously known antimicrobial activity [30, 32, 33], were evaluated against *Candida* spp. planktonic cells and *C. albicans* biofilm. 

As there is no consensus regarding the acceptable inhibition level for natural products when compared with standard antibiotics, we have considered the definition proposed by Duarte et al. [30], being strong activity-MIC up to 0.5 mg/mL; moderate activity-MIC between 0.51 and 1.0 mg/mL, and weak activity-MIC above 1.1 mg/mL. Thus, the results obtained in the present work show a broad action of oils and extracts, with a greater activity for essential oils than for extracts, capable of inhibiting the different *Candida* spp. CBS and clinical isolates with moderate to strong activity. When the plant materials were compared with fluconazole and nystatin activity, the plant materials were similar or better than the aforementioned drugs in some cases. 

Considering the susceptibility of CBS standard strains and the clinical isolates, the strains from CBS collection were, in general, more sensitive to essential oils. Among the different species investigated, clinical isolates from *C. dubliniensis* and *C. tropicalis* were the most resistant species (moderate to weak activities—MIC ≥ 1.0 mg/mL), except in the case of the essential oil from *C. sativum*.

The oil from *C. sativum,* a plant used as a very common condiment in Brazilian Northeast food, was capable of inhibiting all *Candida* spp. CBS and clinical isolates in the initial assays. The fractionation of the oil emphasized *F*_8–10_ fraction activity; however, the application in planktonic cells did not incur in the best activities, indicating the potential use of this plant in the form of crude oil. *F*_8–10_ fractions presented as main constituents 2-hexen-ol, 3-hexen-ol, and 1-decanol, and when tested alone or grouped showed a synergistic action from the compounds, as the mixture presented the lowest MIC values. 

The crude essential oil from *C. sativum* applied at regular intervals affected biofilm development and caused a delay in the lag phase and a decrease in the biofilm growth (Figures 1(b) and 1(c)) when compared to the control assay (Figure 1(a)). The response of *C. albicans* CBS 562 and clinical isolate 13A5 to treatments was different, as this last presented a higher growth in the presence of the drugs and essential oil. The statistical analysis demonstrated a significative difference (*P* ≤ .05) after 48 h of treatment, showing a similar action for nystatin and *C. sativum* essential oil. The activity of the materials tested in the present study was confirmed through SEM analysis and demonstrated cell deformation, presenting a withered appearance. 

## 5. Conclusion

In conclusion, the results of the present study indicate the potential use of themedicinal species for oral candidosis prevention or treatment and emphasize the action of the crude *C. sativum* essential oil, which demonstrates a strong activity against *Candida* spp. planktonic cells and *C. albicans* biofilm. 

## Figures and Tables

**Table 1:** Anti-*Candida* activity (MIC-mg/mL) of the essential oils, the dichloromethane (DE) and hexane (HE) extracts from the medicinal species studied.

*Candida* spp.	*A. tuberosum*		*C. sativum*		*C. martinii*		*C. winterianus*		*S. chamaecyparissus*	
	EO	DE	EO	HE	EO	DE	EO	DE	EO	DE
Culture collection										
*C. albicans *CBS 562	0.500	0.500	0.015	0.125	0.063	0.250	1.000	0.125	1.000	0.250
*C. krusei *CBS 573	>1.0	>1.0	0.015	0.125	0.063	0.125	0.063	0.063	0.500	0.500
*C. parapsilosis *CBS 604	0.015	0.015	0.125	0.031	0.125	0.063	1.000	0.125	1.000	1.000
*C. dubliniensis *CBS 7987	1.000	>1.0	0.007	0.031	0.125	0.250	0.500	0.125	0.063	0.063
*C. tropicalis *CBS 94	>1.0	>1.0	0.125	0.500	1.000	1.000	1.000	1.000	1.000	1.000

Clinical isolates^a^										
*C. albicans *3 A5	0.500	>1.0	0.015	1.000	0.250	1.000	1.000	1.000	1.000	>1.0
*C. albicans *13 A5	0.500	>1.0	0.031	1.000	0.063	1.000	0.500	1.000	1.000	>1.0
*C. albicans *41 M2	0.500	>1.0	0.031	1.000	0.063	1.000	1.000	1.000	1.000	>1.0
*C. albicans *50 M2	0.500	>1.0	0.063	1.000	0.250	1.000	0.500	1.000	1.000	>1.0
*C. krusei *56 M3	0.007	>1.0	0.007	0.031	0.007	0.063	0.031	0.002	0.125	0.063
*C. krusei *56 M4	1.000	>1.0	0.007	0.015	0.007	0.007	0.007	0.007	0.007	0.500
*C. krusei *56 M6	1.000	>1.0	0.007	0.002	0.007	0.007	0.007	0.007	0.007	0.015
*C. parapsilosis *45 M2	0.015	>1.0	0.007	0.063	0.031	0.063	0.250	0.250	0.500	0.125
*C. parapsilosis *45 M4	0.015	>1.0	0.007	0.125	0.015	0.031	0.063	0.063	0.250	0.125
*C. parapsilosis *45 M5	0.015	>1.0	0.015	0.007	0.250	1.000	1.000	0.063	1.000	1.000
*C. dubliniensis *26 A2	1.000	>1.0	0.063	0.250	0.250	0.500	1.000	0.500	0.500	0.500
*C. dubliniensis *26 A3	1.000	>1.0	0.031	0.250	0.250	0.500	1.000	0.250	0.250	0.500
*C. dubliniensis *26 A4	>1.0	>1.0	0.500	0.250	1.000	0.250	1.000	0.250	0.500	1.000
*C. tropicalis *53 M6	>1.0	>1.0	0.125	0.500	>1.0	>1.0	>1.0	>1.0	1.000	1.000
*C. tropicalis *53 M7	>1.0	>1.0	0.063	0.250	0.250	0.250	1.000	1.000	>1.0	>1.0
*C. tropicalis *53 M8	>1.0	>1.0	0.031	0.250	>1.0	>1.0	>1.0	>1.0	>1.0	>1.0

^a^The codes correspond to the number of the volunteers and the place of sample origin; (A)-periodontal pocket; (M) oral mucosa.

**Table 2:** Anti-*Candida* activity (MIC-mg/mL) of crude essential oil (EO) and *C. sativum* fractions (F).

*Candida *spp.	EO	*F*_3_–*F*_4_	*F*_5_	*F*_6_	*F*_7_	*F*_8_–*F*_10_
Culture collection						

*C. albicans* CBS 562	0.015	0.063	>1.0	0.500	0.125	0.015
*C. krusei* CBS 57	0.015	0.500	0.500	0.250	0.125	0.063
*C. parapsilosis* CBS 604	0.125	0.500	>1.0	0.250	0.250	0.063
*C. dubliniensis* CBS 7987	0.007	0.125	>1.0	0.063	0.063	0.031
*C. tropicalis* CBS 94	0.125	0.500	>1.0	1.000	0.063	0.063

Clinical isolates^a^						
*C. albicans* 13 A5	0.031	0.125	>1.0	0.500	0.250	0.063
*C. krusei* 56 M3	0.007	0.250	0.250	0.250	0.125	0.063
*C. parapsilosis* 45 M4	0.007	0.500	>1.0	0.250	0.250	0.063
*C. dubliniensis* 26 A3	0.031	0.250	>1.0	0.250	0.125	0.063
*C. tropicalis* 53 M7	0.063	>1.0	>1.0	1.000	0.250	0.125

^a^The codes correspond to the number of the volunteers and the place of sample origin; (A)-periodontal pocket; (M) oral mucosa.

**Table 3:** Identified compounds, retention time (Rt), retention Index (RI), and relative percentage (%) in crude oil and *C*. *sativum *essential oil fractions *F*_8–10_.

Rt (min)	RI	Compounds	% Relative	
			Crude oil	*F*_8–10_
3.54	—	3-hexen-1-ol <E>	—	0.44
3.63	—	3-hexen-1-ol <Z>	1.24	16.08
3.78	—	2-hexen-1-ol <Z>	0.77	14.52
5.19	933	*α*-pinene	0.15	**—**
10.23	1101	Undecane	0.33	—
10.40	1106	Nonanal	0.28	—
13.02	1172	Nonanol	0.19	—
14.49	1209	Decanal	10.97	—
16.73	1262	2-decenal <E>	2.74	—
17.12	1272	2-decen-1-ol <E>	4.11	—
17.32	1277	1-decanol	15.30	44.16
18.65	1309	Undecanal	2.20	—
20.96	1364	2-undecenal <E>	1.01	—
21.25	1371	2-undecenol <E>	1.21	1.71
21.38	1375	Undecanol	1.96	3.62
22.89	1411	dodecanal	7.53	—
25.19	1469	2-dodecenal	8.16	—
25.45	1475	2-dodecenol	11.26	13.04
25.52	1477	Cyclododecane	2.54	4.43
26.89	1512	Tridecanal	1.00	—
29.11	1570	2-tridecenal <E>	1.44	—
29.28	1574	2-tridecenol <E>	0.86	—
29.56	1581	Caryophyllene oxide	0.47	—
30.78	1614	Tetradecanal	1.26	—
33.01	1674	2-tetradecenol <E>	13.58	—
36.58	1775	2-pentadecenol	3.10	—
40.03	1879	2-hexadecenol	0.80	—
47.73	—	Phytol	3.06	—
—	—	nonidentified compounds	2.52	2.01

**Table 4:** Anti-*Candida* activity (MIC-mg/mL) of the *C. sativum* essential oil (OE), fractions *F*_8–10_ and from the major compounds present in *F*_8–10_.

*Candida *spp.	EO	Fractions *F*_8–10_	2 -hexen-ol cis	2-hexen-ol trans	3-hexen-ol cis	3-hexen-ol trans	1-decanol	Majority compounds mixture
*C. albicans *CBS 562	0.015	0.015	0.063	0.031	0.500	0.063	0.031	0.0005
*C. albicans *13 A	0.031	0.063	0.063	0.007	0.125	0.031	0.015	0.0005
